# Mandibular metastasis as an initial manifestation of hepatocellular carcinoma: A report of two cases

**DOI:** 10.3892/ol.2015.2864

**Published:** 2015-01-12

**Authors:** CHUNHUA DU, YUANYONG FENG, NINGYI LI, KE WANG, SHUANGYI WANG, ZHENHUA GAO

**Affiliations:** 1 Department of Internal Medicine, Affiliated Hospital of Qingdao University, Qingdao, Shandong 266003, P.R. China; 2 Department of Oral and Maxillofacial Surgery, Affiliated Hospital of Qingdao University, Qingdao, Shandong 266003, P.R. China; 3 Department of Oral and Maxillofacial Surgery, Beijing Stomatological Hospital, Capital Medical University, Beijing 100050, P.R. China

**Keywords:** hepatocellular carcinoma, mandible, metastasis

## Abstract

The present study reports two cases of mandibular metastasis from hepatocellular carcinoma (HCC), including the clinical presentation, and computed tomography (CT), histopathology and immunohistochemistry results. Space-occupying lesions occurred unilaterally as an initial mandibular manifestation. HCC metastasis was confirmed by post-surgical examination, and the primary tumor was found using CT scans. Hepatitis B virus infection history and positive results of hepatitis B surface antigen, hepatitis B e-antibody, hepatitis B core antibody and hepatitis B virus pre-S1 antigen further supported the pathogenesis of HCC. Based on the clinical findings, the characteristics of the CT scans and the histopathology and immunohistochemistry results, the mechanisms of HCC metastasis and its management are also discussed.

## Introduction

Metastasis is a step in the development of malignant tumors that is influential with regard to cancer-related patient mortality. As one of the most frequently diagnosed cancers worldwide, hepatocellular carcinomas (HCCs) are prone to metastasize to other tissues (1). The most common sites for extrahepatic metastases are the lung, regional lymph nodes, adrenal gland, bones, brain and peritoneum (2).

With a rich blood supply and a specific growth-promoting microenvironment, bone tissue is the third most common target for metastasis overall, after the liver and lungs (3). In malignant tumors, breast and prostate carcinomas are the most prone to developing bone metastases, followed by lung carcinoma, colorectal cancer, thyroid carcinoma and renal carcinoma (4). Clinically, the incidence of metastasis in HCC is 1.6–16% (5), of which 5–7% of the cases present with bone metastases as the initial lesions. By contrast, for bone metastasis, the axial skeleton is the most common metastatic site, with the vertebrae being the most frequently involved tissues (~37% of cases), followed by the ribs, sternum and pelvis (6). Compared with these sites, mandibular metastases from HCC are rather rare, particularly when they occur with symptoms (7).

The present study reports two cases of HCC metastatic to the mandible, without any other metastases, and describes the treatment for the initial mandibular lesion. Written informed consent was obtained from the patients.

## Case report

### Case one

**Figure 1:**
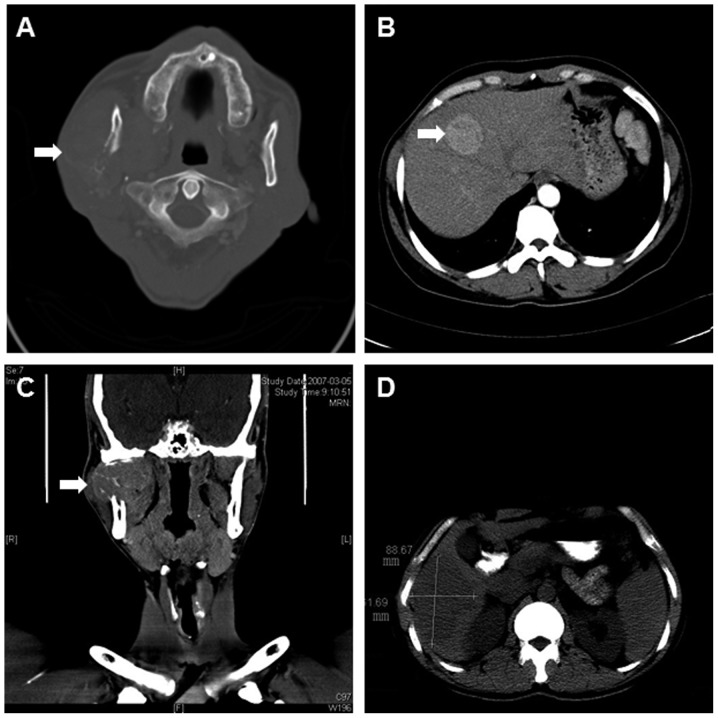
(A and B) Case one: (A) Plain computed tomography scan showing the tumor and osteolytic lesions in the right mandibular ramus. (B) The primary tumor of the hepatocellular carcinoma was identified in the medial segment of the left liver lobe following surgery. (C-D) Case two: (C) Coronal scan showing the tumor in the right mandible, with extension to the cranial base. (D) Primary tumor, 88×78 mm in size and with uneven density, identified in the right liver lobe following surgery.

**Figure 2:**
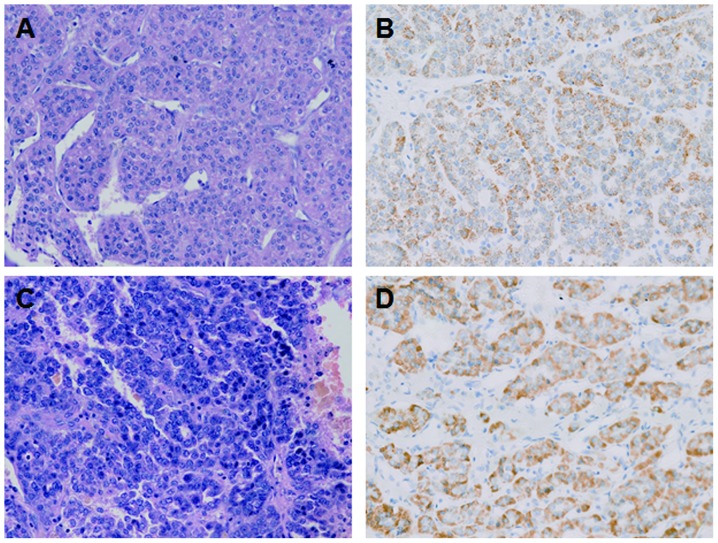
Case one: (A) Histopathology revealing the hepatocellular carcinoma metastasis, with moderate differentiation (magnification, ×100). (B) HAb18, a specific antibody for HCC, was relatively strongly expressed as brown staining in the cytoplasm (magnification, ×200). Case two: (C) Histopathology results revealing the hepatocellular carcinoma in the connective tissues (magnification, ×100). (D) HAb18 was relatively strongly expressed as brown staining in the cytoplasm (magnification, ×200).

A 57-year-old male was admitted to the Affiliated Hospital of Qingdao University (Qingdao, China) in July 2005, presenting with a painless swelling in the preauricular region and numbness of the lower lip for six months. Maxillofacial asymmetry was observed, and the immobile tumor could be localized by palpation in the right subaural and preauricular areas, in the absence of tenderness. Intraoral examination disclosed a soft and smooth mass extending from the right maxillary molar to the tuberosity. Computed tomography (CT) revealed an osteolytic lesion of 4×4.5×5 cm in the right mandibular ramus (Fig. 1A). In addition to a negative chest X-ray, physical examination showed no evident dysfunction of the liver or kidneys. Tests for hepatitis B surface antigen (HBsAg), hepatitis B e-antibody (HBeAb), hepatitis B core antibody (HBcAb) and hepatitis B virus pre-S1 antigen were all positive. The histopathological analysis of a needle biopsy specimen from the tumor showed moderately differentiated metastatic HCC, positive for HAb18 (a specific marker for HCC) in immunohistochemical staining, which was relatively highly expressed in the cytoplasm (Fig. 2A and 2B). Thereafter, enhanced CT scans of the right and left liver lobes further confirmed the presence of a space-occupying lesion, which was mainly located in the upper segment (Fig. 1B). The carcinoma was radically resected, with the surrounding normal tissues, under general anesthesia. Histopathological examination of the resected specimen revealed metastatic HCC. The patient was treated with hepatic infusion chemotherapy (750 mg 5-fluorouracil, 60 mg cisplatin and 40 mg pirarubicin) just once. In the follow-up period, ascites occurred two months after the drug administration, and the overall survival time was one year. The direct cause of mortality was liver failure.

### Case two

A 37-year-old male was admitted to the Affiliated Hospital of Qingdao University in March 2007, presenting with a painless and progressive swelling in the right mandible, initially 1.0×1.0×1.0 cm in size, which had been apparent for three months, and numbness of the lower lip, which had persisted for almost one month. Prior to admission to the Affiliated Hospital of Qingdao University, the patient was fist admitted to the Dental Clinic of Longkou People’s Hospital (Yantai, China) in Nov 2006 for a toothache in the right mandible for four months, which was identified as an accompanying symptom. The patient was first admitted to the Dental Clinic for a toothache in the right mandible, which was actually an accompanying symptom. A physical examination showed a solid 4×4×2.5-cm tumor in the ramus region. Thereafter, CT scans revealed a 6.0×4.8-cm osteolytic lesion in the upper segment of the right mandibular ramus, involving the masseter muscle and the lateral pterygoid (Fig. 1C). Enriched nuclide was observed in the emission CT (ECT) ^99m^Tc-methylene diphosphonate scans. The chest X-ray result was negative. No kidney or liver abnormalities were evident upon physical examination. Tests for HBsAg, HBeAb, HBcAb and hepatitis B virus pre-S1 antigen were all positive.

The needle biopsy of the tumor revealed metastatic HCC in the connective tissues, positive for HAb18 in immunohistochemical staining, which was relatively highly expressed in the cytoplasm (Fig. 2C and 2D). CT scans of the abdomen showed a 88.67×61.69-mm lesion of unequal low density (Fig. 1D) and swelling of the spleen. The carcinoma was radically resected, with the surrounding normal tissues, under general anesthesia. Histopathological examination of the resected specimen revealed metastatic HCC. The patient survived for five months without any medical intervention.

## Discussion

HCC is considered to be the sixth most frequently diagnosed cancer, accounting for 5.7% of all new cancer cases and/or mortalities per year, 82% of which occur in developing countries (55% in China alone) (8). In recent years, the management of HCC has been rapidly improved due to the enhancement of surgical and chemotherapeutic interventions (9). Although the life expectancy of patients with HCC has increased, distant metastasis remains a challenge in the treatment of this disease. Essentially, there are two mechanisms of metastasis from the liver to the maxillofacial territory. The first mechanism involves the hepatic artery and the portal vein. When tumor tissues affect these vessels, metastatic dissemination would reach the lung first and then the maxillofacial area. Alternatively, it has been postulated that there may be a connection between the azygos or hemiazygos veins and the vertebral venous plexus (Batson’s plexus), which creates another route for hematogenous spread. There would consequently be free communication between the neck, thorax, abdomen and pelvis venous systems, and the non-valve vertebral venous plexus, which extends from the cranial base to the coccyx. Any increase in the intra-abdominal pressure can result in an ascendant flow through the vertebral venous plexus (7,10). In such cases, HCC cells could reach the maxillofacial territory through these hematogenous routes and metastasize into the mandible (11).

Mandibular metastasis from HCC depends on the histological grade and the degree of differentiation of the tumor cells, as well as the local anatomy and characteristics of the bone tissues. As previously reported, the main factors affecting metastasis are the structure of the blood vessels and the volume of red bone marrow (12). In the process of metastasis, dissemination of the cells from carcinomas or thrombus mixed with carcinoma cells may contribute to the formation of metastatic lesions. The specific structure of the mandible, lower blood flow rates in the canal and abrupt alteration to this can result in the permanent implantation of a tumor thrombus (13). Additionally, Van der Waal *et al* (14) found that there was an increasing risk for tumor embolus formation with low blood flow in red bone marrow. Up to 25% of the adult mandibular marrow cavity is occupied by red bone marrow, mainly in the regions of the third molars and premolars, which become the principal targets for tumor metastasis.

With regard to oral malignancies, only 1–3% are metastatic carcinomas from distant primary sites; these sites in order of decreasing frequency are the breasts, adrenal gland, colorectal system, genital organs and thyroid gland in females, and the lungs, prostate, kidneys, bones and adrenal gland in males (15). However, metastatic HCC to the mandible is rarely identified (16,17). In the current case report, the presenting symptoms were a painless lump in the ramus and numbness of the lower lip. Upon admittance, the diagnosis was of a malignant mandibular tumor, according to the maxillofacial CT scans. A final diagnosis of HCC metastasis was then formed based on the results from the pathological and immunohistochemical examinations.

Chronic hepatitis B virus (HBV) and/or hepatitis C virus (HCV) infection is the primary risk factor for the development of HCC (18). Following decades of chronic hepatitis infection, 30–40% of patients suffer from liver cirrhosis, 1–5% of which subsequently develop HCC (19). This intimate association between HCC and HBV infection indicates that a liver examination is recommended when patients have a history of hepatitis and/or the result of an HBsAg test is positive upon histopathological analysis. In the present study, the positive results of HBsAg, HBcAb and hepatitis B virus pre-S1 antigen tests were in line with the diagnosis of HCC made by a further liver examination using CT.

The major characteristics of mandibular metastasis are masses with or without toothache, numbness of the lower lip or the involved skin, and dysfunction of the temporomandibular joint for condyle involvement (20). Bleeding is one of the most frequently reported complications of metastases (21). However, no bleeding was observed in either of the present two cases.

As the initial diagnosis in the present study was of a mandibular tumor, which was consequently diagnosed as a metastasis from HCC, the association and differences between primary and secondary tumors requires consideration and verification. Specifically, when significant lumps and/or pain is present at the secondary site, but there are no clear symptoms at the primary site, positron emission tomography-CT and single-photon emission CT may contribute to the evaluation of early metastasis in the bone tissues. Furthermore, histopathological evaluation should be considered for the final diagnosis of potential metastatic carcinomas.

In the present study, two cases of HCC metastasis which had migrated to the mandible, with extension to the ramus and condyle, were identified. Although the articular disc and the cranial bones were found to be unaffected in either case, simultaneous dissection of the tumor and the articular disc was performed to avoid recurrence. In spite of an increased survival time and extended life-expectancy following treatment, the prognosis of extrahepatic metastasis remains unsatisfactory. Therefore, the management of metastatic carcinomas is mainly focused on pain relief and organ preservation. Based on the findings of the present two cases, radical dissection of the secondary tumors combined with arterial infusion chemotherapy was attempted for the primary tumors. However, the survival time of the two cases was less than one year. Accordingly, radiotherapy to control bleeding and/or pain relief should be considered as an essential option to prevent disease progression.
